# Association between vitamin B group supplementation with changes in % flow-mediated dilatation and plasma homocysteine levels: a randomized controlled trial

**DOI:** 10.3164/jcbn.17-56

**Published:** 2019-03-07

**Authors:** Koutatsu Maruyama, Ehab S. Eshak, Minako Kinuta, Masanori Nagao, Renzhe Cui, Hironori Imano, Tetsuya Ohira, Hiroyasu Iso

**Affiliations:** 1 Laboratory of Community Health and Nutrition, Special Course of Food and Health Science, Department of Bioscience, Graduate School of Agriculture, Ehime University, 3-5-7 Tarumi, Matsuyama, Ehime 790-8566, Japan; 2 Public Health, Department of Social Medicine, Osaka University Graduate School of Medicine, 2-2 Yamadaoka, Suita-shi, Osaka 565-0871, Japan; 3 Department of Public Health and Preventive Medicine, Minia University, Main Road, Shalabyland, Minia 61111, Egypt; 4 Department of Public Health, Dokkyo Medical University, School of Medicine, 880 Kita-kobayashi, Mibu, Shimotsuga-gun, Tochigi 321-0293, Japan; 5 Department of Epidemiology, Fukushima Medical University School of Medicine, 1 Hikariga-oka, Fukushima, Fukushima 960-1295, Japan

**Keywords:** folate, vitamin B6, vitamin B12, plasma homocysteine, clinical trial

## Abstract

There is limited evidence examining the association between B vitamin supplementation and improved endothelial function via lowering plasma homocysteine levels. This study investigated whether low-dose B vitamin supplementation improves endothelial dysfunction in Japanese adults with one or more components of metabolic syndrome. A randomized, controlled, crossover trial, without a washout period or blinding of subjects, was conducted from May-September, 2010. The subjects were 127 Japanese men and women aged 40–65 years who had at least one component of metabolic syndrome without medication. Participants took a supplement drink for two months but were divided into early intervention or later intervention groups. The flow-mediated dilatation, plasma homocysteine level, serum B-vitamins, and vitamin C levels were measured. A significant increase in serum B vitamins and vitamin C levels, and a reduction in plasma homocysteine levels were observed. The mean serum homocysteine level pre- and post-intervention was 9.8 and 8.2 µmol/L in the early intervention group and 10.8 and 7.4 µmol/L in the later intervention group (*p*<0.01). However, no significant changes in flow-mediated dilatation was found. Low-dose multivitamin supplementation including B vitamins is associated with a significant reduction in plasma homocysteine levels among patients with one or more components of metabolic syndrome. This study was registered at the University Hospital Medical Information Network (UMIN) centre, and has the identifier UMIN000004436.

## Introduction

Endothelial dysfunction is a key event in the development of atherosclerosis.^(1)^ Homocysteine is a sulphur-containing amino acid generated during the metabolism of the essential amino acid methionine to cysteine. High levels of homocysteine have been shown to induce endothelial injury.^(2)^ An ultrasound assessment of flow-mediated dilatation (FMD) in the brachial artery is an indirect measure of endothelial nitric oxide release and is considered as a surrogate marker for endothelial function.^(3)^ Multivitamin supplementation was associated with improvement of surrogate vascular outcomes of endothelial dysfunction. For example, after vitamin C or a combination of vitamin C and E supplementation, levels of FMD, augmentation index, central aortic pressure,^(4)^ lipid peroxidation,^(5,6)^ and blood pressure^(7)^ were improved. Supplementation of folic acid, alone or combined with other B vitamins, was also associated with improved endothelial function via lowering plasma homocysteine levels,^(8,9)^ or even independent of homocysteine-lowering.^(10)^ However, most previous studies were conducted on patients with coronary artery disease (CAD),^(4,7–10)^ and with a very high supplementation dosage that cannot be obtained normally via diet alone.^(4,5,10)^ Evidence regarding the effects of low-dose supplementation in non-CAD patients on the biomarkers related to endothelial function is limited. Thus, this study investigated whether low-dose B vitamin supplementation would improve endothelial dysfunction in Japanese adults with one or more symptoms of metabolic syndrome.

## Materials and Methods

A randomized, controlled, crossover trial, without a washout period or blinding of subjects, was conducted from May–September 2010. The subjects were volunteers aged 40–65 years who lived around Osaka Prefecture (one of the metropolitan prefectures), who had at least one component of metabolic syndrome according to Japanese criteria,^(11)^ and were non-medicated for hypertension, diabetes or hyperlipidemia in a recent health check-up. Our participants’ characteristics i.e., using vitamin supplementation, smoking status, physical activity, plasma homocysteine levels and serum vitamin B levels, were more or less comparable with those of adult Japanese general population studied in previous reports.^(12–16)^

**Fig. 1:**
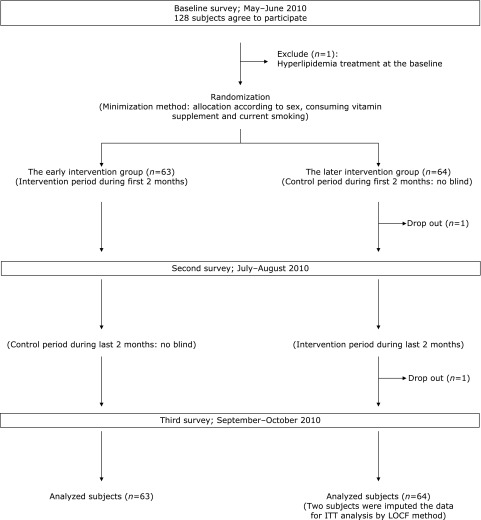
Study design and sampling scheme. The figure shows the timeline and sampling scheme, i.e., randomization, the intervention, and control periods for each group, the number of participant dropout and the analysed subjects in each group, and the methods of the intention-to-treat (ITT) analysis.

Participants were recruited and gave their informed consent which yielded a sample of 89 males and 39 females between the ages 40–65 years. After excluding a subject who had a hyperlipidemia treatment at the baseline survey, the final number of subjects was 88 men and 39 women (Fig. 1). Furthermore, two participants dropped out of the study, and upon analysis, their data were imputed using the Last Observation Carried Forward (LOCF) method. This study was approved by the Ethics Committee of Osaka University Graduate School of Medicine, was registered at the University Hospital Medical Information Network (UMIN) centre, and has the identifier UMIN000004436. According to the study objectives and related previous studies, and considering a power of 80%, α = 0.05 and a 20% dropout rate, a 120-subject sample size (60 in each group) was determined for the study.

The participants were randomly divided into the early intervention or later intervention groups after the baseline survey, using the minimization method assigned by one researcher who did not participate in any measurement. The proportion of sex, use of vitamin supplements (yes/no), and current smoking habits (yes/no) in relation to both the supplementation components and the outcomes of this study were taken into account.

The subjects in the early intervention group were asked to take one pack of the supplement drink every morning during the first two months of the study, while the subjects in the later intervention group were asked to take one pack of the supplement drink every morning for the last two months of the study. The supplement drink was V CRESC BERRIES (125 ml per pack, NUTRI Co., Ltd., Japan): a product marketed as containing vitamin B6, B12, C and folate. The details of the drink contents are shown in Table 1.

The participants recorded whether they had supplement drink or not during the intervention period. They were also asked not to change their usual lifestyles and not to consume any supplements including vitamin B6, B12, folate, and anti-oxidative components during both the intervention and the control periods. In both groups, the data were collected at baseline, two months, and four months, and changes in each outcome were analysed.

FMD was determined using high-resolution ultrasonography and a forearm occlusive cuff by two well-trained observers. Participants were asked to refrain from smoking and exercise before the health examination. They were also required to fast at least six hours or more before the measurements were taken. The details of the measurements are described in a previous study.^(17)^ In brief, high-resolution ultrasound with a 10-MHz linear array transducer (UNEX Co. Ltd., Nagoya, Japan) was used to record longitudinal images of the right brachial artery at baseline and continuously from 30 s before to at least 2 min after cuff deflation. Computer-assisted analysis software (UNEX Co. Ltd., Nagoya, Japan) was used to determine the diameter of the brachial artery semi-automatically as previously described.^(18)^ A baseline longitudinal image of the artery was acquired for 30 s, and then the blood pressure cuff was inflated to 50 mmHg above systolic pressure for 5 min. FMD was expressed as the percentage change from baseline as follows: %FMD = (brachial artery diameter at hyperemia – brachial artery diameter at baseline)/brachial artery diameter at baseline × 100. The determination of endothelial function was performed in accordance with published guidelines.^(17)^ A previous study reported that the intra-class correlation coefficient was 0.84–0.99 for intra-observer reproducibility and 0.82–0.87 for inter-observer reliability.^(19)^

Biomarkers in blood samples, anthropometry, and blood pressure levels were measured to confirm health status, including metabolic syndrome, for all subjects. Serum triglycerides, glucose, and high-density lipoprotein (HDL)-cholesterol were measured by the Osaka Medical Centre for Health Science and Promotion, an international member of the US National Cholesterol Reference Method Laboratory Network (CRMLN).^(20)^ The serum triglycerides level was also measured enzymatically, and the serum total cholesterol and HDL-cholesterol levels were measured enzymatically by an automatic analyser (Hitachi 7250, Hitachi Medical Corp., Hitachi, Japan). The serum glucose level was measured by the hexokinase method using the same instrument.

Furthermore, several blood biomarkers levels related to the supplementation in this study were measured by an SRL, Inc., Tokyo, Japan. Plasma of total homocysteine and serum vitamin B6 and C levels were measured using YMC-UltraHT Pro C18 (YMC CO., LTD., Tokyo, Japan), and Wakosil-II 5C18HG (Wako Pure Chemical Industries, Ltd., Osaka, Japan) on a high performance liquid chromatography (HPLC) system (Shimadzu Corporation, Kyoto, Japan). Serum vitamin B12 and folate were measured using Access Vitamin B12 and Access Folate (Beckman Coulter Inc, Fullerton, CA) by Chemiluminescent Enzyme Immuno Assay (CLEIA) (UniCel DxI 800, Beckman Coulter Inc, Fullerton, CA).

Height and weight were measured in light clothing to determine body mass index (BMI). BMI is calculated using the following formula: weight (kg)/[height(m)]^2^. Overweight was defined as BMI≥25 kg/m^2^. Waist circumference was measured by tape measure at the level of the umbilicus and the symphysis pubis at the maxim protrusion of the hips, while in a standing position and breathing normally. Blood pressure was measured using an automatic sphygmomanometer three times in the sitting position after resting for a few minutes. Only the third measurement of blood pressure was used for each participant. Metabolic syndrome was defined according to Japanese criteria^(11)^ as: abdominal obesity (waist circumference ≥85 cm for men and ≥90 cm for women) and any two of the following three factors: high blood pressure (systolic blood pressure ≥130 mmHg and/or diastolic blood pressure ≥85 mmHg), high fasting blood glucose (≥6.1 mmol/L), and dyslipidemia (serum triglycerides ≥1.7 mmol/L and/or HDL-cholesterol <1.03 mmol/L).

Lifestyle was assessed at every health examination. Trained public health nurses asked subjects about their alcohol habits (current/ex-/non-drinker), smoking habits (current/ex-/non-smoker), engagement in regular physical exercise for 15 min or more per week, and medical treatment for major chronic diseases. Trained dieticians also asked the participants about their dietary habits during the previous month to estimate dietary intake using validated comprehensive and brief self-administered diet history questionnaires.^(21,22)^ All nutrient and food group intakes were adjusted for energy intake by a density method.

Analysis of variance for a crossover design was performed for each variable, using the general linear model. The subject effects, carryover effects (a term for intervention received in the previous intervention period), intervention effects (supplement vs no supplement), and period effects were tested in a two-sided test with a 5% level of significance. All statistical analyses were performed with SAS software (ver. 9.4; SAS Institute Inc., Cary, NC).

## Results

Table 2 shows the baseline characteristics of the early and later intervention groups. The proportion of men was approximately 70% in both groups. Age and other baseline characteristics did not significantly differ between the two groups, except for the proportion of those with abdominal obesity and high blood glucose. However, there were no observed differences in major outcomes (i.e., %FMD, homocysteine and serum Vitamin B groups and C) between the two groups.

**Fig. 2:**
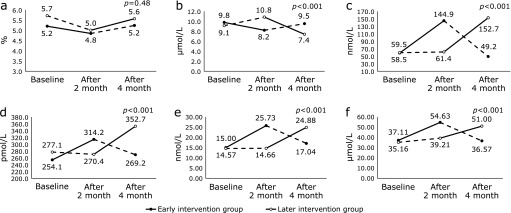
Changes in %FMD, plasma homocysteine, and serum vitamin B groups and C levels in the early and later intervention groups. The black circle denotes the early intervention group. The open circle denotes the later intervention group. The straight line denotes the intervention period. The dotted line denotes the non-intervention period for (a) %FMD, (b) plasma homocysteine, (c) serum vitamin B6 (pyridoxal), (d) serum vitamin B12, (e) serum folate, and (f) serum vitamin C levels. *P* values denote the effect of the intervention.

Table 3 and Fig. 2 show each outcome measured and their changes during the intervention and control periods for the early and later intervention groups at baseline, two months, and four months. There was a significant reduction in plasma homocysteine levels associated with the intervention. The mean plasma homocysteine level at baseline, two months, and four months was 9.8, 8.2, and 9.5 µmol/L, respectively, in the early intervention group, and 9.1, 10.8, and 7.4 µmol/L, respectively, in the later intervention group. There were also significant increases in serum vitamin B6, B12, C, and folate levels associated with the intervention (*p*<0.001).

There were no significant changes in %FMD or the components of metabolic syndrome associated with the intervention except for increased serum triglycerides levels.

There were also no significant carryover effects on the plasma homocysteine levels, i.e., no intervention received in the previous intervention period (*p*>0.10: not shown in the table). However, significant carryover effects on serum vitamin levels (*p*<0.10) were found. Additionally, no harm was done to any subject during this trial.

When we conducted stratification analysis by sex, we observed significant effects of vitamin supplementation on plasma homocysteine and serum vitamins but not on %FMD for both sexes (data were not shown). Furthermore, when we restricted the analyses to subjects with a certain component of metabolic syndrome; such as dyslipidemia or high blood pressure, we did not find any significant reduction in the %FMD (data were not shown).

## Discussion

The present study demonstrated that oral multivitamin supplementation containing B vitamins for two months is associated with significant reductions in plasma homocysteine levels, significant increases in serum vitamin B6, B12, C and folate levels, but no significant changes in %FMD.

The significant increases of vitamin B6, B12, C and folate levels after intervention were caused by the supplementation for 2 months. The half-life of vitamin B6, B12, C and folate are 25 days,^(23)^ 6 days,^(24)^ 10–20 days,^(25,26)^ and 100–200 days,^(27,28)^ respectively. Thus, the timing of blood drawing unlikely affected the results.

Previous studies among CAD patients have shown that folate supplementations at doses ranging from 0.127–10 mg/day were associated with homocysteine levels that were reduced by 3.7% to 34%, with varying degrees of reduction for individual doses.^(29–32)^ The Homocysteine Lowering Trialists’ meta-analysis examined the effect of folate supplementation on plasma homocysteine levels among 1,114 subjects across 12 trials, and showed that dietary folic acid reduced blood homocysteine concentrations by 25%, with similar effects of folate supplementation in the range of 0.5–5 mg daily.^(33)^ The results of the present study fall midway between the higher percentages of reduction seen in this meta-analysis^(33)^ and the lower percentages of reduction seen in patients with CAD.^(31)^

Two studies have shown that the magnitude of plasma homocysteine-lowering is dependent upon baseline homocysteine concentrations and upon folate pre-treatment concentrations.^(33,34)^ The baseline plasma homocysteine and folate levels of participants in the present study were 9.5 µmol/L and 6.5 µmol/L, respectively. These results are similar to those of CAD patients in a Portland study, in which the baseline homocysteine and folate levels were 9.9 µmol/L and 6.3 µmol/L, respectively. The Portland study also showed a 3.7%, 11%, and 14% reduction in homocysteine levels after five-weeks of folate supplementation in doses of 0.127 mg/day, 0.499 mg/day, and 0.655 mg/day, respectively.^(31)^ Therefore, comparatively, the modest reduction in homocysteine levels (13–19%) found in subjects without severe cardiovascular risk in the present study is considered a reasonable effect of low dose vitamin B supplementation on plasma homocysteine levels.

The biological mechanisms by which each vitamin of the vitamin B complex (folate and other vitamin B) contributed to the reduction of plasma homocysteine levels have been investigated. Not only folic acid but also vitamin B12 is an essential cofactor for the remethylation of homocysteine to methionine leading to reduction on plasma homocysteine levels.^(33)^ Dietary folic acid reduced homocysteine levels by 25% and vitamin B12 produced an additional 7% reduction in blood homocysteine levels, whereas vitamin B6 did not have any significant additive effect.^(35)^

In the current study, B vitamin supplementation reduced the plasma levels of homocysteine without significant changes in the %FMD or the components of metabolic syndrome except for serum triglycerides. Similar to the present results, Lonn *et al.*^(8)^ reported that supplements with B vitamins lowered homocysteine levels by 2.2 µmol/L, but did not reduce the risk of cardiovascular events.

Compared with the conventional atherosclerotic risk factors, the influence of plasma homocysteine levels on the carotid intimal-medial thickness was negligible.^(36)^ Doshi *et al.*^(10)^ reported that folate improved the endothelial function via mechanisms largely independent of homocysteine-lowering. A folate dose of 5 mg/day among CAD patients was associated with a 9.3% reduction in homocysteine levels (from 10.6 µmol/L to 8.3 µmol/L) and with improved FMD from 52.5 µm to 111 µm. However, there were no correlations found between the improvement of FMD and the reduction in homocysteine levels.^(10)^ Moreover, the folate-induced improvement of endothelial function was observed acutely, 2 h after the first oral dose of folate, before any significant reduction in homocysteine levels were detected.^(10)^ In another study, using 5 mg/day of folate for 6 weeks, plasma homocysteine levels were reduced from 11.1 µmol/L to 9.3 µmol/L, and the FMD improved from 52 µm to 110 µm; however, again, there were no correlations between the two changes.^(37)^

The supplement drink in this study contained vitamin D, C, E vitamins in addition to B vitamins. According to several studies, the effects of multivitamin supplementation on cardiovascular disease (CVD) risk factors are controversial.^(4,38–44)^ Therapies that contain vitamin C alone,^(38,39)^ or with vitamin E,^(40)^ were reported to prevent acute impairment in endothelial function. In addition, long-term vitamin C supplementation has been signiﬁcantly associated with FMD improvement in patients with CAD.^(4,41)^ On the other hand, a recent systematic review and meta-analysis of 16 randomized controlled trials found no effect of vitamin D on endothelial function,^(42)^ and two large-scale trials could not find any association between vitamin C and E supplements and major cardiovascular outcomes.^(43,44)^

Title *et al.*^(30)^ found that a supplement containing folic acid and other antioxidants (vitamin C and E) did not signiﬁcantly improve endothelial function, whereas a supplement containing folate alone did, despite the beneﬁcial effects of combined therapy on lipid peroxidation. Some unfavourable interactions between vitamins C, E, and folate might modify the effects of vitamin B supplementation. This possible modification was confirmed by Ashor *et al.*^(45)^ who reported significant improvements in endothelial function in trials supplementing with vitamin C alone and vitamin E alone. However, co-administration of both vitamins C and E was ineffective.

Additionally, it is worth mentioning that at each examination, most of the participants in this study did not have enough fasting that serum triglycerides levels were likely to be raised.^(46)^ Therefore, the result of serum triglycerides should be interpreted carefully.

The limitations of the present study warranted for discussion. First, the *in vivo* interaction of combined supplementation cannot be concluded, as the sole effect of each of B vitamins, vitamin C, or vitamin E was not evaluated. Second, a washout period was not applied in this study, which caused the carryover effect on serum vitamins B6 and B12, folate, and vitamin C. However, there was no significant carryover effect of the plasma homocysteine level. Third, a blind technique was not applied in this study. However, the mean dietary intakes of vitamins B6 and B12, folate, and vitamin C assessed by BDHQ at two and four months were similar to those at baseline for both the early and later intervention groups (data were not shown). Thus, dietary intakes of these vitamins during the study period were unlikely to be affected by the intervention.

In conclusion, among subjects with one or more components of metabolic syndrome, low-dose multivitamin supplementation including B vitamins was associated with a significant reduction in plasma homocysteine levels, but not with %FMD.

## Figures and Tables

**Table 1:** Contents of supplement drink for this study

Retinol, µg	300.0
Retinol equivalents, µg	300.0
Vitamin B1, mg	3.0
Vitamin B2, mg	3.0
Vitamin B6, mg	5.0
Vitamin B12, µg	10.0
Vitamin C, mg	500.0
Niacine, mg	15.0
Folate, µg	800.0
Vitamin D3, µg	5.0
Vitamin E, mg	20.0
Biotin, µg	50.0
Pantothenic acid, mg	10.0
α-Lipoic acid, mg	30.0

**Table 2:** Baseline characteristics

	Early intervention group	Later intervention group	*p* value*
*N*	63	64	
Age, year	53.9 (6.1)	53.2 (6.8)	0.52
Men, *n* (%)	44 (69.8)	44 (68.8)	0.89
Supplementation of vitamin B6, B12 and folate, *n* (%)	4 (6.4)	4 (6.3)	1.00
Habitual physical activity, *n* (%)	41 (65.1)	36 (56.3)	0.31
Current smoker, *n* (%)	10 (15.9)	13 (20.3)	0.52

Abdominal obesity, *n* (%)	61 (96.8)	50 (78.1)	<0.01
High blood pressure, *n* (%)	48 (76.2)	52 (81.3)	0.49
High blood glucose, *n* (%)	25 (39.7)	15 (23.4)	0.05
Dislipidemia, *n* (%)	46 (73.0)	44 (68.8)	0.60
Metabolic syndrome, *n* (%)	48 (76.2)	39 (60.9)	0.06

FMD, %	5.2 (2.5)	5.7 (2.5)	0.25

Plasma homocysteine, µmol/L	9.8 (3.9)	9.1 (4.0)	0.36
Serum vitamin B6 (pyridoxal), nmol/L	58.5 (96.1)	59.5 (75.8)	0.95
Serum vitamin B12, pmol/L	254.1 (146.1)	277.1 (127.2)	0.35
Serum folate, nmol/L	15.00 (7.18)	14.57 (6.61)	0.72
Serum vitamin C, µmol/L	37.11 (16.26)	35.16 (13.55)	0.46

Total energy intake, kcal	1,935 (560)	1,940 (595)	0.96
Vitamin B6 intake, mg/1,000 kcal	0.6 (0.1)	0.6 (0.2)	0.80
Vitamin B12 intake, µg/1,000 kcal	4.5 (2.3)	4.7 (2.4)	0.70
Folate intake, mg/1,000 kcal	166.5 (52.6)	171.1 (63.7)	0.66
Vitamin C intake, mg/1,000 kcal	54.5 (23.8)	56.3 (30.9)	0.72

SD or percentage in parentheses. *Student’s *t* test or chi square test.

**Table 3:** The mean (SD) at each examination and changes of FMD, plasma homocysteine and serum vitamins concentration of early and later intervention groups

	Early intervention group				Later intervention group				Changes		
	Baseline	After intervention (2 months)	After control (4 months)		Baseline	After control (2 months)	After intervention (4 months)		Intervention period	Control period	*p* value*
FMD, %	5.2 (2.5)	4.8 (2.7)	5.2 (3.3)		5.7 (2.5)	5.0 (2.3)	5.6 (2.6)		0.10	−0.16	0.48
Waist circumference, cm	95.7 (6.4)	94.0 (6.9)	94.6 (7.3)		93.6 (6.9)	91.5 (6.8)	92.8 (7.0)		−0.32	−0.66	0.50
HDL–cholesterol, mmol/L	1.20 (0.25)	1.14 (0.23)	1.20 (0.21)		1.22 (0.25)	1.17 (0.26)	1.22 (0.25)		0.00	0.00	0.71
Triglycerides, mmol/L	2.45 (1.19)	2.78 (1.63)	2.56 (1.35)		2.57 (1.84)	2.37 (1.24)	2.66 (1.93)		0.31	−0.21	0.03
Serum glucose, mmol/L	6.30 (1.64)	6.25 (2.48)	6.26 (1.67)		5.70 (1.00)	5.64 (0.67)	5.67 (0.89)		−0.02	−0.02	0.97
Systolic blood pressure, mmHg	138.4 (16.0)	136.2 (16.8)	135.5 (15.0)		137.0 (15.9)	134.3 (16.4)	137.2 (16.0)		0.29	−1.69	0.27
Diastolic blood pressure, mmHg	88.0 (9.7)	84.1 (12.0)	84.5 (11.0)		87.3 (11.0)	83.0 (10.4)	86.1 (11.2)		−0.41	−1.99	0.18
Plasma homocysteine, µmol/L	9.8 (3.9)	8.2 (1.9)	9.5 (4.6)		9.1 (4.0)	10.8 (8.0)	7.4 (1.9)		−2.50	1.51	<0.001
Serum vitamin B6 (pyridoxal), nmol/L	58.5 (96.1)	144.9 (90.6)	49.2 (71.6)		59.5 (75.8)	61.4 (68.4)	152.7 (76.4)		88.8	−46.9	<0.001
Serum vitamin B12, pmol/L	254.1 (146.1)	314.2 (156.7)	269.2 (107.8)		277.1 (127.2)	270.4 (112.0)	352.7 (177.9)		71.2	−25.8	<0.001
Serum folate, nmol/L	15.00 (7.18)	25.73 (7.79)	17.04 (6.21)		14.57 (6.61)	14.66 (7.65)	24.88 (8.11)		10.47	−4.30	<0.001
Serum vitamin C, µmol/L	37.11 (16.26)	54.63 (18.19)	36.57 (12.48)		35.16 (13.55)	39.21 (11.47)	51.00 (12.89)		14.65	−7.00	<0.001

SD in parentheses. *Intervention effect.
